# Blind Source Separation of Hemodynamics from Magnetic Resonance Perfusion Brain Images Using Independent Factor Analysis

**DOI:** 10.1155/2010/360568

**Published:** 2010-04-21

**Authors:** Yen-Chun Chou, Chia-Feng Lu, Wan-Yuo Guo, Yu-Te Wu

**Affiliations:** ^1^Department of Biomedical Imaging and Radiological Sciences, National Yang-Ming University, No. 155, Section 2, Li-Nong Street, Pei-Tou, Taipei 112, Taiwan; ^2^Integrated Brain Research Laboratory, Department of Medical Research and Education, Taipei Veterans General Hospital, No. 201, Section 2, Shih-Pai Rd., Pei-Tou, Taipei 112, Taiwan; ^3^Department of Radiology, Taipei Veterans General Hospital, No. 201, Section 2, Shih-Pai Rd., Pei-Tou, Taipei 112, Taiwan; ^4^Faculty of Medicine, National Yang-Ming University, No. 155, Section 2, Li-Nong Street, Pei-Tou, Taipei 112, Taiwan; ^5^Institute of Brain Sciences, National Yang-Ming University, No. 155, Section 2, Li-Nong Street, Pei-Tou, Taipei 112, Taiwan

## Abstract

Perfusion magnetic resonance brain imaging induces temporal signal changes on brain tissues, manifesting distinct blood-supply patterns for the profound analysis of cerebral hemodynamics. We employed independent factor analysis to blindly separate such dynamic images into different maps, that is, artery, gray matter, white matter, vein and sinus, and choroid plexus, in conjunction with corresponding signal-time curves. The averaged signal-time curve on the segmented arterial area was further used to calculate the relative cerebral blood volume (rCBV), relative cerebral blood flow (rCBF), and mean transit time (MTT). The averaged ratios for rCBV, rCBF, and MTT between gray and white matters for normal subjects were congruent with those in the literature.

## 1. Introduction

Dynamic susceptibility-contrast perfusion magnetic resonance (MR) imaging is a commonly used method for the noninvasive assessment of cerebral blood perfusion because of the availability of MR imaging units and lack of exposure to ionizing radiation [1–10]. After a bolus injection of intravascular contrast agent, the passage of bolus induces the susceptibility inhomogeneity, which in turn causes a relative decrease of image intensities of brain tissues from the baseline. Various tissues manifest distinct blood-supply patterns since the contrast agent arrives consecutively, leading to temporal intensity drops at different time instants. Based on the perfusion data, we can calculate the hemodynamic parametric maps, that is, relative cerebral blood volume (rCBV), relative cerebral blood flow (rCBF), and mean transit time (MTT), by employing the indicator dilution theory [11, 12]. This has been used in the assessment of many brain disorders such as tumors [2, 7, 13], stroke [14], infection [15], and moyamoya disease [16, 17].

The estimation of rCBV and rCBF, however, requires arterial concentration of the contrast agent as an arterial input function (AIF). This is a demanding task, and many methods have been proposed to address the issue. Additionally, classification of blood-supply patterns for various tissue types in brain based on bolus transit profiles is essential for the assessment of brain perfusion. Wiart et al. [18] manually selected single or multiple pixels for the tissue of interest and used the averaged temporal profile as a reference in producing a similarity map for segmenting gray matter (GM) and white matter (WM) from perfusion images. This method is advantageous for easy implementation but limited to extraction of a single tissue type per similarity map. If one attempts to segment multiple tissue types, associated similarity maps need to be created one by one after cumbersome selections of reference pixels, which may be prone to operator influence. Alternatively, Martel et al. [19] used the conventional factor analysis, under the hypothesis that the observable signal-time curve for each pixel is a weighted sum of pure physiological factors, to classify the signal-time curves of arterial and venous structures from MR dynamic perfusion images. This method has also been applied in nuclear medicine to separate cardiac components and extract left ventricular input function from dynamic H_2_^15^O PET images [20–22]. It should be noted that, although the factor-analysis-based methods are attractive, additional assumptions of a priori knowledge are required to obliquely rotate the eigenvectors to yield meaningful physiological factors [23–25]. Besides, only one or two physiological factors were resolved in past studies. To our knowledge, the automatic simultaneous segmentation of multiple perfusion compartments has been less explored. A recent related work was to use the expectation-maximization algorithm with a mixture of multivariate Gaussian models for fitting the perfusion MR images so that each pixel can be labeled by the resulting maximal posterior probability [26].

The aim of this study is to automatically classify the spatiotemporal blood-supply patterns which enables us to extract the arterial compartment for modeling the AIF and segment other tissue regions as well. To this end, we employ an independent factor analysis (IFA) [27], a data-driven method, allowing us to blindly separate mixed signals into independent-factor (or independent-source) components for multitissue hemodynamic classification. That is, the hemodynamics of each tissue type can be dissected without making a priori spatial and temporal assumptions of physiology. The factors in the IFA, in contrast to the conventional factor analysis, are modeled by a finite mixture of Gaussian functions that can be used as a constraint to remove rotational factors [27]. This method has been applied to successfully separate the background factors and noise artifacts from the stimulus-evoked MEG and EEG sensor data contaminated by large background brain activity [28]. In this study, the use of IFA is based on two inherent assumptions: (1) signal intensity of each pixel is a linear mixture contributed from different tissues, referred to as the partial volume mixing, which is a well-known phenomenon due to finite resolution of MR scan; (2) the anatomic structures of pure tissue types are spatially independent (nonoverlapped with each other). The classification of multitissue hemodynamics consists of two steps. The first step is to identify a dominant tissue type on each independent-factor (IF) image resolved from the IFA based on the arrival order of corresponding signal-time curve and a priori knowledge of brain anatomy. The second step is to automatically extract regions of the dominant tissue from each selected IF image by means of an optimal threshold [29].

This paper is organized as follows. The protocol of MR imaging and data recordings is first described followed by the introduction of IFA method and calculation of the pixel-by-pixel rCBV, rCBF, and MTT maps. Computer simulations are presented to validate the application of IFA method on two-dimensional independent factors. Resultant five IF images in conjunction with corresponding signal-time curves from a data set are exhibited. We then calculate and display the rCBV, rCBF, and MTT maps based on the extracted arterial compartment. The averaged ratios for rCBV, rCBF, and MTT between GM and WM from five normal subjects are also computed. Finally, we discuss and conclude this study.

## 2. Subjects and Data Recording

**Figure 1:**
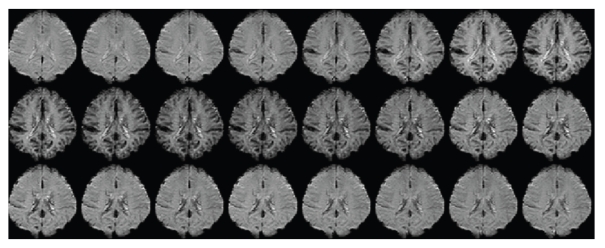
Perfusion MR images from a 39-year-old volunteer. Images encompass the first circulation (7–30, from left to right, top to bottom) at an upper slice location.

Five healthy volunteers (three males and two females) aged from 18 to 47 were recruited to participate in this study. Written informed consent was obtained from each volunteer before this study. A multislice gradient-echo EPI pulse sequence on a 1.5-Tesla scanner (Signa CV/i; GE Medical Systems, Milwaukee, WI, USA) was used and the imaging parameters were transaxial imaging, TE/TR  =  60/1000 milliseconds, flip angle  =  90 degrees, FOV = 24 cm × 24 cm, matrix = 128 × 128, slice thickness/gap  =  5/5 mm for 7 slices, one acquisition, and 100 images per slice location. Twenty milliliters of Gd-DTPA-BMA (Omniscan, 0.5 mmol/mL; Nycomed Imaging, Oslo, Norway) followed by 20 mL of normal saline were delivered administratively by a power injector (Spectris; Medrad, Indianola, PA, USA) at a flow rate of 3-4 mL/sec in the antecubital vein. The first thirteen and last thirty-seven images were removed from 100 images and fifty images, which exhibited stable baseline and discernible temporal signal changes, were kept in a slice location for analysis. The temporal resolution is one second. Figure 1 displays part of dynamic perfusion images at an upper slice location encompassing the first circulation from a 39-year-old volunteer. With 16384 (=128 × 128) pixels for each image, the observation of 50 temporal images can be represented by a 50 × 16384 matrix. The signal-to-noise ratios (SNRs) of these five data sets were 51, 55, 77, 83, and 88, respectively.

## 3. Independent Factor Analysis

In this study, we assume that there are *N* pure tissue types presented on perfusion images and that signal intensity of each pixel is a linear combination of contributions from *N* pure tissues due to the effect of partial volume mixing in MR scanning. Let the observed noisy mixtures be denoted by a matrix **y** of size 50 × 16384, the linear mixing by a matrix **H** of size 50 × *N*, the independent factors by a matrix **x** of size *N* × 16384, and the zero-mean Gaussian noise by a matrix **u** of size 50 × 16384, where each row in **x** represents an image for a tissue type, each column of **H** represents a signal-time curve for an associated tissue type, and covariance matrix of the zero-mean Gaussian noise is denoted by Λ. Under the assumption that the sources **x** are mutually statistically independent, the IFA-based blind source separation (BSS) technique attempts to recover the unknown mixing matrix **H** and hidden IF images **x** from the observed noisy mixtures [27]:

(1) y=Hx+u.

In order to recover the IF images, we assume that each row of the matrix **x** is a realization of a random variable whose probability density *p*(*x*_*j*_) is in a form of mixture of Gaussians (MoG) given by

(2) $$\begin{array}{cc} p\left(x_{j}\;|\;\theta_{j}\right) & =\overset{n_{j}}{\underset{q_{j}=1}{\sum }}\omega_{j,q_{j}}g\left(x_{j}-\mu_{j,q_{j}},\nu_{j,q_{j}}\right)\\ & =\overset{n_{j}}{\underset{q_{j}=1}{\sum }}\omega_{j,q_{j}}\frac{1}{\sqrt{2\pi \nu_{j,q_{j}}}}\exp\;\left[-\frac{1}{2}\frac{{\left(x_{j}-\mu_{j,q_{j}}\right)}^{2}}{\nu_{j,q_{j}}}\right],\\ \theta_{j} & =\left\{\omega_{j,q_{j}},\mu_{j,q_{j}},\nu_{j,q_{j}}\right\}, \end{array}$$

where *g* stands for the Gaussian function, *q*_*j*_ is a variable with value running over number of Gaussians with means *μ*_*j*,*q*_*j*__, variances *ν*_*j*,*q*_*j*__ used in MoG model for the source *x*_*j*_, *j* = 1,…, *N*, and *ω*_*j*,*q*_*j*__'s are the mixing proportions satisfying ∑_*q*_*j*__ *ω*_*j*,*q*_*j*__ = 1. Let **q** = (*q*_1_,…, *q*_*N*_) denote all possible combinations of the individual *q*_*j*_. The joint probability density *p*(**x**) in *N*-dimensional space can be formed by the product of the one-dimensional MoG's in (2) due to the mutual independence of *x*_*j*_, which is itself an MoG

(3) $$p\left(x\;|\;\theta \right)=\overset{N}{\underset{j=1}{\prod }}p\left(x_{j}\;|\;\theta_{j}\right)=\underset{q}{\sum }‍\omega_{q}g\left(x-\mu_{q},V_{q}\right),$$

where

(4) $$\begin{array}{cc} \omega_{q} & =\overset{N}{\underset{j=1}{\prod }}\omega_{j,q_{j}}\\ & =\omega_{1,q_{1}},\ldots ,\omega_{N,q_{N}},\\ \mu_{q} & =\left(\mu_{1,q_{1}},\ldots ,\mu_{N,q_{N}}\right),\\ V_{q} & =\mathrm{diag}\;\left(\nu_{1,q_{1}},\ldots ,\nu_{N,q_{N}}\right),\\ g\left(x-\mu_{q},V_{q}\right) & =\overset{}{\underset{j}{\prod }}g\left(x_{j}-\mu_{j,q_{j}},\nu_{j,q_{j}}\right),\\ \sum_{q} & =\sum_{q_{1}}\ldots \sum_{q_{N}}. \end{array}$$

In our implementation, *n*_*j*_ was determined to be 2 for each source *x*_*j*_. To estimate the IF model parameters, the Kullback-Leibler distance function [27] is employed to measure the difference between the probability density of the observed signals given the model parameters **W**, that is, *p*(**y** | **W**), and the observed one, that is, *p*°(**y**), which is given by

(5) $$\begin{array}{cc} \varepsilon \left(W\right) & =\int p^{\circ }\left(y\right)\log\;\frac{p^{\circ }\left(y\right)}{p\left(y\;|\;W\right)}dy\\ & =-E\left[\log\;p\left(y\;|\;W\right)\right]-H_{p^{\circ }}, \end{array}$$

where **W** = {**H**, Λ, ***θ***} denotes the unknown mixing matrix **H**, noise covariance Λ, and MoG parameters ***θ***. The operator *E* denotes the average over the observed **y**. The first term in (5) is the negative log-likelihood of the observed signals given the model parameters **W**, and the second term is the entropy of observed signals, which is independent of **W** and will henceforth be dropped. Note that, based on *p*(**q**, **x**, **y** | **W**) = *p*(**q**)*p*(**x** | **q**)*p*(**y** | **x**), *p*(**x** | **q**) = *g*(**x** − ***μ***_**q**_, **V**_**q**_), and *p*(**y** | **x**) = *g*(**y** − **H** **x**, Λ), the probability density *p*(**y** | **W**) can be expressed in a closed form:

(6) $$p\left(y\;|\;W\right)=\underset{q}{\sum }\int p\left(q\right)p\left(x\;|\;q\right)p\left(y\;|\;x\right)dx=\underset{q}{\sum }p\left(q\right)p\left(y\;|\;q\right)$$

where *p*(**y** | **q**) = *g*(**y** − **H** ***μ***_**q**_, **H** **V**_**q**_ **H** ^*T*^ + Λ). By minimizing *ε*(**W**) with respect to **W** based on the expectation-maximization (EM) method and denoting **W**′ the parameters obtained from the previous iteration, the iterative algorithm can be summarized as follows [27].

1. Initialize all the unknown parameters, that is, **W** ^0^ = {**H** ^0^, Λ^0^, ***θ*** ^0^}.
2. Compute the conditional mean of
  (7) $$\begin{array}{cc} \langle x^{T}\;|\;y,W^{\prime }\rangle & =\int \;\;x^{T}p\left(x\;|\;y,W^{\prime }\right)dx,\;\\ \langle {xx}^{T}\;|\;y,W^{\prime }\rangle & =\int {xx}^{T}p\left(x\;|\;y,W^{\prime }\right)dx,\\ \langle x_{j}\;|\;q_{j},y,W\prime \rangle & =\int x_{j}p\left(x_{j}\;|\;q_{j},y,W^{\prime }\right)dx_{j},\;\\ \langle x_{j}^{2}\;|\;q_{j},y,W\prime \rangle & =\int x_{j}^{2}p\left(x_{j}\;|\;q_{j},y,W^{\prime }\right)dx_{j},\\ & \;p\left(q_{j}\;|\;y,W^{\prime }\right). \end{array}$$
3. Update the mixing matrix and noise covariance:
  (8) $$\begin{array}{cc} H & =E\left[y\langle x^{T}\;|\;y,W^{\prime }\rangle \right]{\left(E\left[\langle {xx}^{T}\;|\;y,W^{\prime }\rangle \right]\right)}^{-1},\\ \Lambda & =E\left[{yy}^{T}\right]-E\left[y\langle x^{T}\;|\;y,W^{\prime }\rangle H^{T}\right]. \end{array}$$
4. Update the source MoG parameters:
  (9) $$\begin{array}{cc} \mu_{j,q_{j}} & =\frac{E\left[p\left(q_{j}\;|\;y,W^{'}\right)\langle x_{j}\;|\;q_{j},y,W\prime \rangle \right]}{E\left[p\left(q_{j}\;|\;y,W^{\prime }\right)\right]},\;\\ \nu_{j,q_{j}} & =\frac{E\left[p\left(q_{j}\;|\;y,W^{\prime }\right)\langle x_{j}^{2}\;|\;q_{j},y,W^{\prime }\rangle \right]}{E\left[p\left(q_{j}\;|\;y,W^{\prime }\right)\right]}-\mu_{j,q_{j}}^{2},\\ \omega_{j,q_{j}} & =E\left[p\left(q_{j}\;|\;y,W^{\prime }\right)\right]. \end{array}$$
5. Rescale the parameters to cancel the extra freedom of scaling for facilitating convergence:
  (10) $$\begin{array}{c} \sigma_{j}^{2}=\overset{n_{j}}{\underset{q_{j}=1}{\sum }}\omega_{j,q_{j}}\left(\nu_{j,q_{j}}+\mu_{j,q_{j}}^{2}\right)-{\left(\overset{n_{j}}{\underset{q_{j}=1}{\sum }}\omega_{j,q_{j}}\mu_{j,q_{j}}\right)}^{2},\\ \mu_{j,q_{j}}⟵\frac{\mu_{j,q_{j}}}{\sigma_{j}},\;\;\nu_{j,q_{j}}⟵\frac{\nu_{j,q_{j}}}{\sigma_{j}^{2}},\;\;H_{ij}⟵H_{ij}\sigma_{j}. \end{array}$$
6. Repeat (2)–(5) until the error function *ε*(**W**) converges.

Finally, the IF images can be reconstructed from the estimated model parameters **W** and measured data *y* using the least-mean-square estimator:

(11) $${\hat{x}}^{\text{LMS}}\left(y\right)=\underset{q}{\sum }p\left(q\;|\;y\right)\left(A_{q}y+b_{q}\right),$$

where *p*(**q** | **y**) = *p*(**q**)*p*(**y** | **q**)/∑_**q**′_ *p*(**q**′)*p*(**y** | **q**′), **A**_**q**_ = (**H** ^*T*^Λ^−1^ **H** + **V**_**q**_^−1^)^−1^ **H** ^*T*^Λ^−1^, and **b**_**q**_ = (**H** ^*T*^Λ^−1^ **H** + **V**_**q**_^−1^)^−1^ **V**_**q**_^−1^ ***μ***_**q**_.

## 4. Calculation of the rCBV, rCBF, and MTT

Prior to the calculation of pixel-by-pixel rCBV, rCBF, and MTT maps, we computed the concentration-time curve *C*_*t*_(*t*) for each pixel using the formula

(12) $$C_{t}\left(t\right)=-\frac{k}{\text{TE}}\ln\;\left(\frac{S\left(t\right)}{S_{0}}\right),$$

where *k* is an unknown constant, TE is the echo time, and *S*(*t*) and *S*_0_ are the signal intensities of each pixel at time *t* and at the baseline, respectively [1, 4, 5, 30]. By using the indicator dilution theory, one can determine the rCBV for each pixel as a ratio of the area integrating over the first pass (e.*g*., from 1st to 22nd images in Figure 1) of the contrast agent under the concentration-time curve, *C*_*t*_(*t*), to that under the AIF, *C*_*a*_(*t*) [11, 12],

(13) $$\text{rCBV}=\frac{\int_{\text{first}\;\text{pass}}^{}c_{t}\left(t\right)dt}{\;\int_{\text{first}\;\text{pass}}^{}c_{a}\left(t\right)dt},$$

where *C*_*a*_(*t*) is the concentration-time curve for the arterial region. The rCBF can be computed based on the relationship with concentration-time curve for each pixel [31]:

(14) $$C_{t}\left(t\right)=\text{rCBF}\cdot C_{a}\left(t\right)⊗R\left(t\right),$$

where ⊗ denotes convolution, · denotes multiplication, and *R*(*t*) is the residue function for the pixel. The rCBF · *R*(*t*) curve for each pixel in (14) can be resolved using the singular value decomposition (SVD) method and the value of rCBF at each pixel was determined by the maximum value of rCBF · *R*(*t*) curve [1]. Finally, the MTT of contrast-agent particles passing through a pixel was defined to be [1, 4, 5]

(15) $$\text{MTT}=\frac{\text{rCBV}}{\text{rCBF}}.$$

## 5. Results

**Figure 2:**
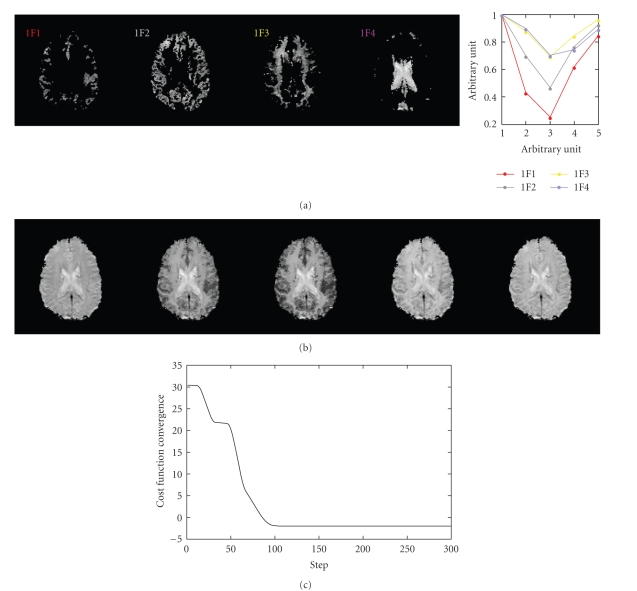
Computer simulation for the evaluation of the IFA method. (a): The four hypothetical IF images and columns of the hypothetical mixing matrix (the right most plot). (b): The simulated images. (c): The convergence of the IFA algorithm.

In order to validate the implementation of IFA algorithm, a computer simulation was conducted before perfusion data were processed. Since the purpose of this simulation was to verify applicability of the IFA method on the separation of two-dimensional IF images, rather than validate the theory of IFA method (readers are referred to [27] for detailed computer simulations), we simply created four hypothetical IF images (128 × 128) with zero background and foreground related to four major tissue types, that is, artery (IF1: 603 pixels), GM (IF2: 1683 pixels), WM (IF3: 1514 pixels), and others (IF4: 924 pixels including vein, sinus, choroid plexus, and cerebral spinal fluid) (the left four plots in Figure 2(a)). In practice, the four tissue-related areas were segmented from the perfusion data and copied to the corresponding locations. The corresponding averaged temporal profile of each area during the first pass was simplified into five points to generate the hypothetical mixing matrix **H** (5 × 4) (also see the right most plot in Figure 2(a))

(16) $$H=\left[\begin{array}{cccc} 1 & 1 & 1 & 1\\ 0.4324 & 0.6992 & 0.8789 & 0.8975\\ 0.2529 & 0.4676 & 0.6940 & 0.7026\\ 0.6186 & 0.7655 & 0.8457 & 0.7438\\ 0.8464 & 0.9269 & 0.9636 & 0.8934 \end{array}\right].$$

We arranged the four IF images into a matrix **x** (4 × 16384) which was multiplied by the **H** to create a matrix **y** = **H** **x** (5 × 16384). Additional multivariate Gaussian random noises with zero mean and diagonal covariance matrix (diagonal terms were 10) were added to the noise-free simulated data **y** (**y** is displayed in Figure 2(b)). The SNR was 240.

Now the task was to estimate four IF images and mixing matrix from the matrix **y**, to compare them with the hypothetical ones. The number *n*_*j*_ of Gaussian functions in the MoG model was chosen to be 2 for modeling the probability density function of each IF image and minimizing the computational cost since we have experienced that the use of larger *n*_*j*_ did not improve the results in either simulated or perfusion data. The values of cost function (the first term in (5)) reduced quickly and converged after around 100 iterations (Figure 2(c)). The correlation value between each pair of hypothetical and estimated IF images and that between each pair of hypothetical and estimated mixing weightings were higher than 0.9999. We further computed a matrix $J={\left({\hat{H}}^{T}\hat{H}\right)}^{-1}{\hat{H}}^{T}H$ , where $\hat{H}$ was the estimated mixing matrix, and the result was

(17) $$J=\left[\begin{array}{cccc} 0.9982 & -0.0016 & -0.0011 & 0.0015\\ 0.0042 & 1.0030 & 0.0019 & -0.0044\\ -0.0041 & -0.0029 & 1.0002 & 0.0029\\ 0.0017 & 0.0014 & -0.0010 & 1.0001 \end{array}\right],$$

which was very close to the identity matrix if the estimate was correct. We repeated the simulation when the SNR was reduced to 40, which was lower than that in the normal data. Results showed that the correlation value between each pair of hypothetical and estimated mixing weightings remained as high as 0.9998 and that between each pair of hypothetical and estimated IF image only degraded slight to between 0.9883 and 0.9997. The values of matrix **J** were

(18) $$J=\left[\begin{array}{cccc} 1.0168 & -0.0070 & -0.0010 & 0.0201\\ -0.0106 & 1.0226 & 0.0008 & -0.0462\\ -0.0230 & -0.0096 & 1.0432 & 0.0390\\ 0.0168 & -0.0062 & -0.0432 & 0.9870 \end{array}\right].$$

The high correlation values and good approximation of **J** matrices not only validated the correctness and convergence of our implementation of the IFA method, but also promised the suitability of the IFA method in segregating various tissue types from perfusion MRI data.

**Figure 3:**
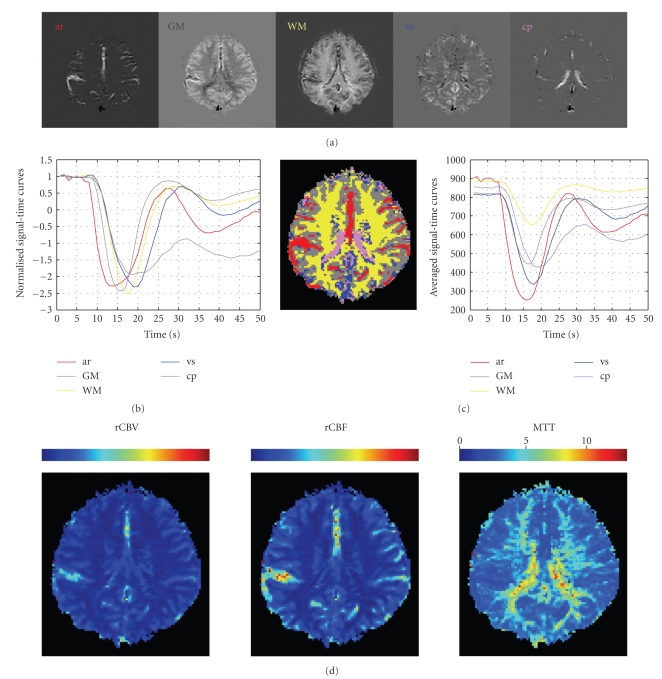
Classification results of a normal subject (whose perfusion images are shown in Figure 1). (a) Five major tissue types exhibited in IF images are ar, GM, WM, vs, and cp, respectively. (b) The normalized signal-time curves correspond to five IF images, where the one (red) with the fastest signal drop is the artery. (c) Left: a color-coded composite map is used to represent the final segmentation result for different tissue types. Right: five signal-time curves correspond to the averaged intensities of color-coded areas. (d) The rCBV map (left), the rCBF map (middle), and the MTT map (right). (Scale unit for rCBV and rCBF: arbitrary unit; scale unit for MTT: second.)

The number of IF images (*N*), that is, number of tissue types, needs to be determined prior to the IFA process. Various numbers, ranging from 4 up to 6, have been assumed in the calculation and we have found that *N* = 5 elucidated distinctly discernible tissue types, appearing to agree with our knowledge of brain anatomy and physiology. Results from five normal image data sets have been confirmed by a neuroradiologist who is one of the coauthors in this study with expertise in perfusion imaging. One of the results was shown in Figure 3, where the upper panel depicts the resultant five IF images, which are artery (ar), GM, WM, vein and sinus (vs), and choroid plexus (cp), respectively. Figure 3(b) displays the corresponding signal-time curves (columns of **H** **)**, respectively, which were all normalized to unit variance and their baselines were shifted to 1.0 for the comparison. The tissue types of IF images were identified based on their anatomical structures and the arrival order of contrast agent, that is, artery follows by GM, WM, vs, or cp. Among all IF images, the IF image with brighter pixels representing artery can be easily recognized because of its signal-time curve presenting the fastest signal drop (red curve in Figure 3(b)). The pixels of each major tissue type can be further segmented out from each IF image using an automatically optimal thresholding technique, that is, the Otsu's method in this study. The final segmentation result was depicted by a color-coded composite image (left panel in Figure 3(c)) and the averaged signal-time curve within each colored area, that is, each tissue type, was computed (right panel in Figure 3(c)).

To create the hemodynamic parametric maps, namely, rCBV, rCBF, and MTT shown in the left, middle, and right panels in Figure 3(d), respectively, we first converted the averaged arterial signal-time curve and temporal intensity profile at each pixel into the concentration-time curves using (12) followed by (13), (14), and (15).

From the segmentation results of the five normal data sets, we have observed that arterial areas were all reliably segmented and the contrast agent consistently arrived first at the artery, followed by GM, WM, vs, and cp. The averaged ratios for rCBV, rCBF, and MTT between GM and WM were 2.139 ± 0.190, 2.598 ± 0.184, and 0.789 ± 0.098, respectively, which were congruent with those in the literature [3, 4, 9, 13].

## 6. Discussion

This study describes an IFA-based method to classify pixels of the same tissue type on perfusion images based on bolus transit profiles and the assumptions of spatial independence as well as the partial-volume mixing effect. The IFA method is flexible in learning the source densities from observed data so that sources can be more accurately modeled by the mixture of Gaussians for the facilitation of subsequent separation. The IFA technique is related to projection pursuit [32, 33], where “interesting” projections of multidimensional data are pursued for optimal visualization of data and exploratory data analysis. Projection pursuit is usually performed by finding directions in which the data is least Gaussian distributed. Since the independent factors in the IFA are modeled by mixture of Gaussian functions, it is interesting to investigate whether the independent factors present maximal non-Gaussian clustering structures in future work. Our results indicated that the brighter pixels in a cluster were homogeneous in most tissues and can be segmented out using Otsu's method for automatic determination of optimal thresholds [29]. Otsu's method is simple in terms of implementation and computation and is robust to histogram irregularities caused by noise.

The determination of the number *N* of IF images is an important issue to be addressed. As suggested by Attias [27], one can determine this number using a comprehensive method, for example, the model comparison method [34], or a simpler but less precise method, for example, the number of significant eigenvalues of data covariance matrix. In this study, we decided *N* based on a priori knowledge of the brain anatomy and arrival order of blood flows for different tissues. When *N* was chosen to be five, each IF image exhibited one dominating tissue cluster with brighter intensities, allowing consistent and reliable segregation of the artery, GM, WM, vs, and cp from the normal subjects. When *N* was less than five, two major tissue types, for examples, artery with GM or vs with cp, can appear at one of IF images. On the other hand, if *N* was larger than five, either one major tissue type was split into two IF images, for example, part of sinus was separated from vs, or repeatedly exhibited at two IF images.

It is worthy to note that the number of unknown parameters depends on the number of dynamic MR images. The more images were used, the more parameters needed to be estimated in the noise covariance Λ and in the mixing matrix **H** in which columns corresponded to the signal-time curves. We have found that the data points encompassed the duration of the first and second circulations, for example, 50 in the illustrative example (Figure 3), were adequate to resolve the IF images. A simple way to determine data length is to average each perfusion image and plot the averaged images with respect to time. The curve delineating the first and second passes is readily seen and the duration can be easily decided.

## 7. Conclusions

The IFA-based method provides several advantages for cerebral hemodynamic studies. First, various tissue compartments on perfusion images can be classified systematically. Second, the arterial compartment can be modeled consistently on the same slice location for calculation of rCBV, rCBF, and MTT maps. Third, bolus transit profiles of these tissues can be well separated, providing information in addition to rCBV, rCBF, and MTT maps, such as temporal scenarios and recirculation of contrast agent. This method also promises differentiation of pathological and nonpathological blood-supply patterns in future clinical applications.

## References

[1] Østergaard L, Weisskoff RM, Chesler DA, Gyldensted G, Rosen BR (1996). High resolution measurement of cerebral blood flow using intravascular tracer bolus passages. Part I: mathematical approach and statistical analysis. *Magnetic Resonance in Medicine*.

[2] Østergaard L, Sorensen AG, Kwong KK, Weisskoff RM, Gyldensted C, Rosen BR (1996). High resolution measurement of cerebral blood flow using intravascular tracer bolus passages. Part II: experimental comparison and preliminary results. *Magnetic Resonance in Medicine*.

[3] Calamante F, Thomas DL, Pell GS, Wiersma J, Turner R (1999). Measuring cerebral blood flow using magnetic resonance imaging techniques. *Journal of Cerebral Blood Flow and Metabolism*.

[4] Rempp KA, Brix G, Wenz F, Becker CR, Gückel F, Lorenz WJ (1994). Quantification of regional cerebral blood flow and volume with dynamic susceptibility contrast-enhanced MR imaging. *Radiology*.

[5] Rosen BR, Belliveau JW, Vevea JM, Brady TJ (1990). Perfusion imaging with NMR contrast agents. *Magnetic Resonance in Medicine*.

[6] Schreiber WG, Gückel F, Stritzke P, Schmiedek P, Schwartz A, Brix G (1998). Cerebral blood flow and cerebrovascular reserve capacity: estimation by dynamic magnetic resonance imaging. *Journal of Cerebral Blood Flow and Metabolism*.

[7] Sorensen AG, Tievsky AL, Østergaard L, Weisskoff RM, Rosen BR (1997). Contrast agents in functional MR imaging. *Journal of Magnetic Resonance Imaging*.

[8] Van Osch MJP, Vonken EJ, Wu O, Viergever MA, Van der Grond J, Bakker CJ (2003). Model of the human vasculature for studying the influence of contrast injection speed on cerebral perfusion MRI. *Magnetic Resonance in Medicine*.

[9] Wenz F, Rempp K, Brix G (1996). Age dependency of the regional cerebral blood volume (rCBV) measured with dynamic susceptibility contrast MR imaging (DSC). *Magnetic Resonance Imaging*.

[10] Wu O, Østergaard L, Weisskoff RM, Benner T, Rosen BR, Sorensen AG (2003). Tracer arrival timing-insensitive technique for estimating flow in MR perfusion-weighted imaging using singular value decomposition with a block-circulant deconvolution matrix. *Magnetic Resonance in Medicine*.

[11] Lassen NA, Perl W (1979). *Tracer Kinetic Methods in Medical Physiology*.

[12] Zierler KL (1962). Theoretical basis of indicator-dilution methods for measuring flow and volume. *Circulation Research*.

[13] Aronen HJ, Glass J, Pardo FS (1995). Echo-planar MR cerebral blood volume mapping of gliomas. Clinical utility. *Acta Radiologica*.

[14] Sorensen AG, Copen WA, Østergaard L (1999). Hyperacute stroke: simultaneous measurement of relative cerebral blood volume, relative cerebral blood flow, and mean tissue transit time. *Radiology*.

[15] Ernst TM, Chang L, Witt MD (1998). Cerebral toxoplasmosis and lymphoma in aids: perfusion MR imaging experience in 13 patients. *Radiology*.

[16] Yamada I, Himeno Y, Nagaoka T (1999). Moyamoya disease: evaluation with diffusion-weighted and perfusion echo-planar MR imaging. *Radiology*.

[17] Ohashi K, Fernandez-Ulloa M, Hall LC (1992). SPECT, magnetic resonance and angiographic features in a moyamoya patient before and after external-to-internal carotid artery bypass. *Journal of Nuclear Medicine*.

[18] Wiart M, Rognin N, Berthezene Y, Nighoghossian N, Froment JC, Baskurt A (2001). Perfusion-based segmentation of the human brain using similarity mapping. *Magnetic Resonance in Medicine*.

[19] Martel AL, Moody AR, Allder SJ, Delay GS, Morgan PS (2001). Extracting parametric images from dynamic contrast-enhanced MRI studies of the brain using factor analysis. *Medical Image Analysis*.

[20] Ahn JY, Lee DS, Lee JS (2001). Quantification of regional myocardial blood flow using dynamic H_2_
^15^O PET and factor analysis. *Journal of Nuclear Medicine*.

[21] Hermansen F, Ashburner J, Spinks TJ, Kooner JS, Camici PG, Lammertsma AA (1998). Generation of myocardial factor images directly from the dynamic oxygen-15-water scan without use of an oxygen-15-carbon monoxide blood-pool scan. *Journal of Nuclear Medicine*.

[22] Wu HM, Hoh CK, Choi Y (1995). Factor analysis for extraction of blood time-activity curves in dynamic FDG-PET studies. *Journal of Nuclear Medicine*.

[23] Barber DC (1980). The use of principal components in the quantitative analysis of gamma camera dynamic studies. *Physics in Medicine and Biology*.

[24] Di Paola R, Bazin JP, Aubry F (1982). Handling of dynamic sequences in nuclear medicine. *IEEE Transactions on Nuclear Science*.

[25] Houston AS (1984). The effect of apex-finding errors on factor images obtained from factor analysis and oblique transformation (nuclear medicine). *Physics in Medicine and Biology*.

[26] Wu YT, Chou YC, Guo WY, Yeh TC, Hsieh JC (2007). Classification of spatiotemporal hemodynamics from brain perfusion MR images using expectation-maximization estimation with finite mixture of multivariate gaussian distributions. *Magnetic Resonance in Medicine*.

[27] Attias H (1999). Independent factor analysis. *Neural Computation*.

[28] Nagarajan SS, Attias HT, Hild KE, Sekihara K (2006). A graphical model for estimating stimulus-evoked brain responses from magnetoencephalography data with large background brain activity. *NeuroImage*.

[29] Otsu N (1979). A threshold selection method from gray-level histograms. *IEEE Transactions on Systems, Man, and Cybernetics*.

[30] Weisskoff RM, Zuo CS, Boxerman JL, Rosen BR (1994). Microscopic susceptibility variation and transverse relaxation: theory and experiment. *Magnetic Resonance in Medicine*.

[31] Kety SS (1960). Blood-tissue exchange methods: theory of blood-tissue exchange and its application to measurement of blood flow. *Methods in Medical Research*.

[32] Friedman JH (1987). Exploratory projection pursuit. *Journal of the American Statistical Association*.

[33] Fyfe C (1997). A comparative study of two neural methods of exploratory projection pursuit. *Neural Networks*.

[34] MacKay DJC (1992). Bayesian interpolation. *Neural Computation*.

